# Real-Life Social-Skills Training and Motor-Skills Training in Adults With Autism Spectrum Disorder: The Con-Tatto Project Walking Down the Francigena Route

**DOI:** 10.3389/fpsyt.2022.846619

**Published:** 2022-04-28

**Authors:** Roberto Keller, Fabio Ardizzone, Caterina Finardi, Rosa Colella, Carmen Genuario, Manuel Lopez, Luana Salerno, Emanuela Nobile, Giovanni Cicinelli

**Affiliations:** ^1^Adult Autism Center, Mental Health Department ASL Città di Torino, Turin, Italy; ^2^Social Services, Unione Net, Settimo Torinese, Italy; ^3^INS Institute of Neuroscience, Florence, Italy

**Keywords:** autism spectrum disorder ASD, motor skill, social skill, depression, social stigma

## Abstract

Autism spectrum disorder (ASD) is a neurodevelopmental disorder with an early onset and a genetic and epigenetic component. ASD is characterized by deficits in socio-emotional reciprocity, impaired verbal and non-verbal communication skills, and specific difficulties in developing and maintaining adequate social relationships with peers. Indeed, restricted, repetitive patterns of behavior, interests, or activities are required by DSM-5 diagnostic criteria. Autistic people usually need an unchanging environment (or in any case predictable and stable) and may have hypo- or hyper-sensitivity to sensory inputs. The onset of clinical symptoms occurs during the early years of life. Social skills competence is a significant therapeutic aim to be pursued when addressing ASD core symptoms. Several considerable motor difficulties (87%) in people with autism spectrum disorder in adulthood have been found. The Con-tatto project developed a project addressing social, physical, and mental health difficulties in real-life walking down the Francigena route for 9 days with 12 autistic people, by (1) Implementing daily sessions of social skills training program whose abilities were addressed to be immediately generalized and used throughout the day. (2) Educational movement and walking activity programs were led by a fitness coach. (3) The creation of walking peers’ social community with a strong and relevant impact on adults with ASD social life respecting every person’s individuality. (4) Provision of social reinforcers to reduce the stigma of people with autism and the experienced perception of low self-esteem, especially when they are bullied.

## Introduction

Autism spectrum disorder (ASD) is a neurodevelopmental disorder with an early onset and a genetic and epigenetic components. ASD is characterized by deficits in socio-emotional reciprocity, impaired verbal and non-verbal communication skills, and specific difficulties in developing and maintaining adequate social relationships with peers. Indeed, restricted, repetitive patterns of behavior, interests, or activities are required by DSM-5 (1) diagnostic criteria. Autistic people usually need an unchanging environment (or in any case predictable and stable) and may have hypo- or hyper-sensitivity to sensory inputs. The onset of clinical symptoms occurs during the early years of life.

Social skills competence is a significant therapeutic aim to be pursued when addressing ASD core symptoms. This complex system of abilities is a demanding goal to achieve for people with ASD (1), especially when they reach adulthood. This weakness in social skills domain is not due only to cognitive impairment but rather to the paucity of evidence-based programs during the developmental age that address social communication and neuroatypical relationship affecting individual functioning (2). Adults with ASD are more likely to reach the adulthood with low functioning in everyday life and in social communication skills because of the absence of ecological and comprehensive habilitation programs conducted during childhood and adolescence. In fact, an analysis carried out on a large sample of autistic adults showed that the interventions provided at developmental age were mainly non-specific psychomotor and speech therapy interventions, if not the total lack of treatment (3). Furthermore, improving social communication and daily life functioning skills during adulthood in an outpatient setting often leads to a lack of generalization across facilities and people, so these abilities are not maintained over time (4).

A recent study highlighted that, other than core symptoms, secondary symptoms need to be considered by clinicians in adults with ASD, such as motor skill deficits (5). Several considerable motor difficulties (87%) in people with autism spectrum disorder in adulthood have been found. These problems range from an uncommon gait to problems with writing (6). Moreover, people with autism spectrum disorder (ASD) are usually not exposed to physical training as much as their typically developing peers (7–9). According to the functional test assessment, many students will need some extra type of support within their typical day to reach consistent exercise levels (10). Finally, ASD or comorbidities are connected to a higher risk of deterioration in motor functioning abilities (11). These findings suggest that the physical domain must be addressed when targeting autism core symptoms. Habilitation processes also lack proper attention to the body. Even though young adults with autism need to perform daily motor and physical activity, this area is often not adequately addressed. In fact, despite the essential role of walking and other motor skills in daily life, a real postural and movement analysis is not taken into consideration in the evaluation of a young adult with ASD (12). Focusing on the general mental health domain, an increased risk of health complications and psychiatric disorders is documented in people with ASD (13). Typically, people with ASD are three times more likely to be diagnosed with depression and four times more likely to suffer from anxiety disorders than healthy peers (Kaiser Permanent in Oakland, California; 14, 15). Moreover, adults with ASD seem to have a higher risk of psychiatric comorbidities such as anxiety, depression, and attention deficit hyperactivity disorder.

## Aim and Hypotheses

The Contatto project developed a project addressing social, physical, and mental health difficulties by:

(1) Implementing daily session of social skills training program whose abilities were addressed to be immediately generalized and used throughout the day. Indeed, we expected a qualitative increase in social skills observed by trainers and participants.
(2) Educational movement and walking activity programs were led by a fitness coach. Therefore, we expected an improvement in all the physical test parameters.
(3) The creation of walking peers’ social community with a strong and relevant impact on adults with ASD social life respecting every person’s individuality.
(4) Provision of social reinforcers to reduce the stigma of people with autism and the experienced perception of low self-esteem, especially when they are bullied. A decreased level of perceived depression and anxiety was expected after the route.

## Materials and Methods

### Participants

Fourteen adult people with autism were originally recruited for the study. All people with ASD, with ASD levels 1 and 2 according to DSM 5 criteria and already known by the Regional Center for Adults with Autism were included; the unique exclusion criteria were the comorbidity with aggressive and/or self-injuring behavior. During the preparation phase two participants decided not to be involved anymore in the project, one due to separation anxiety and the other because of a new job. Therefore, the final sample was composed of twelve people (*n* = 12, *M* = 23,5, DS = 5,4). In total, nine out of twelve participants were diagnosed both with autism and cognitive impairment (5 with mild and 4 with moderate cognitive impairment), and three had autism without cognitive impairment. In total, ten participants were male and two females, six were living in a rural area while the other six were in an urban area. All the participants were assessed by the Regional Center for Adults with Autism in the Piedmont Region before the beginning of the path. All the diagnoses were made according to DSM-5 (1) criteria considering clinical anamnesis, clinical interview, cognitive assessment with WAIS-IV (16), diagnostic evaluation with ADI-R (17) and ADOS module 4 (18) or RAADS (19). Program participation was free of charge and all participants signed informed consent. All the participants were provided with uniforms and several clothes with the project logo, to improve the ability to program the use of the clothes during the route. Moreover, each participant was equipped with a rubberized bracelet with two group leaders’ emergency telephone numbers. No participant used the psychotropic drug during the project. At the end of the project a film was realized (Sul sentiero blu/on the blue way by Gabriele Vacis).

### Experimental Measures

After participants’ recruitment, a psychological and motor skills assessment was led. Thereafter, the first phase included a year-long training route and by the end of this phase, another physical and motor skills assessment was conducted. The last, and second part of the program included nine days walking route. A post-route psychological and motor skills assessment was conducted in order to evaluate post-intervention effects.

For the motor skills evaluation, The Functional Movement Screen [FMS; (20)] tool was used. The FMS is a screening instrument used to evaluate seven fundamental movement patterns in individuals with no current pain complaint or musculoskeletal injury. The FMS is not intended to diagnose orthopedic problems, but rather to demonstrate opportunities for improved movement in individuals. The screen is designed to place an individual in extreme positions where movement deficits become noticeable if appropriate stability and mobility are not used. For research purposes, we used five out of seven movement patterns assessments. Specifically, all the participants were tested with (1) deep squat, (2) inline lounge, (3) shoulder mobility, (4) active straight leg raise, and (5) rotary stability.

We also used the sit and reach test (21) as a measure of flexibility of the lower back and hamstring muscles. Moreover, The Queens College Step test was used as a measure of cardiorespiratory or endurance fitness (22). The test is composed of two parts, the first one is meant to measure the heartbeat when the person is steady and before 3 min of physical activity. In the second part, the heart rate is measured after the subject tries to move up and down the step for 15 s trying to follow the rhythm imposed by the metronome.

The STAI-Y-1 and the STAI-Y-2 (23) were used for the anxiety evaluation. The STAI-Y-1 consists of 20 items that evaluate how the participants feel “right now, at this moment.” STAI-Y-2 scale consists of 20 items that assess how the participant feels “generally.” All the items are scored on a 4-point Likert scale.

Beck Depression Inventory 2nd Edition was used for depression assessment. The scale is composed by 21 items evaluating cognitive, somatic, and emotional aspects of depression (24).

The 23-item PedsQL 4.0 Generic Core Scales Young Adult Version was used to assess participants’ quality-of-life. The PedsQL is composed by 4 domains: (1) physical functioning (eight items); (2) emotional functioning (five items); (3) social functioning (five items); and (4) work/school functioning (five items). The PedsQL 4.0 Young Adult Version was designed as a self-report measure for ages 18–25 years (25).

Regarding the motor skills and psychological assessment, all participants were tested before the first phase of the motor training. Moreover, 1 week before the beginning of the second phase which included the 1-week route, the motor skills of the entire sample population were re-evaluated. Eventually, the motor skills and psychological evaluation was conducted 1 week after the second phase as a post-intervention assessment.

### Treatment

The first phase of the project included a year-long physical training, with a fitness coach with a degree in physical training science. The treatment consisted of 3–4 h of walking route around the Turin area. The training program was at first monthly, then fortnightly, and in the last 2 months once a week. At first, walking routes were made in the urban areas, then in a rural environment with a progressive increase of the kilometers starting from 10 km up to 20 km in the last training sessions with an increase of approximately 2 km per month. The training sessions lasted from March 2020 to May 2021.

The second phase included a walking route for 235 km along paths and roads down to the Francigena route in May–June 2021. The route was divided into nine stages with an average of 26 km per day of walking. The route started from Proceno (on the border between Tuscany and Lazio) and continued until Piazza San Pietro in Rome.

The leaders’ team were a psychiatric doctor (Head of the Regional Center for Adults with Autism in the Piedmont Region), two educators, a nurse, a fitness coach, and four volunteers from the Rotary Club District 2031. A film documentation team was present, previously authorized by the participants and family members in order to film every part of the route.

**FIGURE 1:**
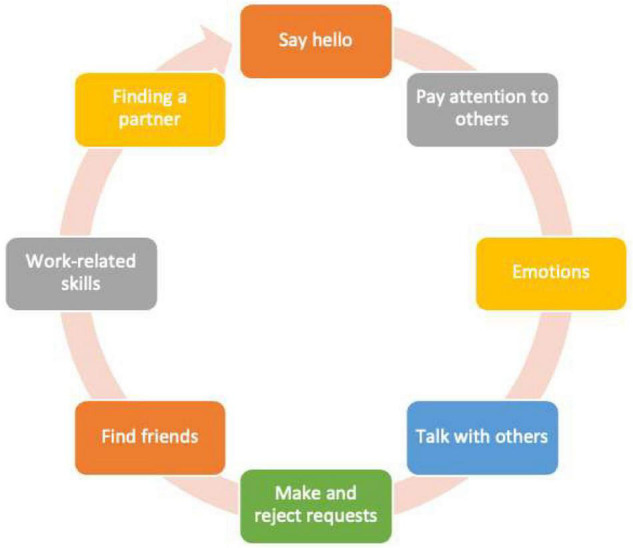
Social skills training program.

A manualized social skills training program was run daily during the route (26). The social abilities table is presented in Figure 1. The sessions were held approximately 2 h per day depending upon the situations and places (hostels, parks, and open countryside). A behavioral skill training (27) procedure was used in order to teach all social abilities. The method consists of several steps: instructions, modeling, rehearsal, and feedback. Indeed, role-playing was used as a key technique to let participants experience and try the newly learned abilities. Generalization was encouraged across participants, places, and people. The group conductor was always the same and all participants have always joined the sessions. During the project, the participants received verbal prompts and social reinforcement all day long from trainers.

The leaders group lived with participants 24 h per day. The latter were walking together, eating together, and sleeping together with the leaders at night.

Throughout the journey, the group was accompanied by the local guides and followed by a car for any support needs. In fact, the car was equipped with an advanced emergency medical kit, whereas the doctor (psychiatrist) and the nurse of the group had an emergency and first aid kit with themselves. The psychiatrist had specific training for remote rescue.

The group was welcomed by the local public authorities in most of the cities it went through. Indeed, in Rome, the group met parliamentarians, state senators, and the Minister for Disabilities and had a private audience with Pope Francis.

The path and the project were broadcasted on a dedicated Facebook page (link).

## Results

Descriptive analyses of psychological and motor tests of the entire sample are presented in Table 1. Motor performances were assessed three times while the psychological aspects only at the beginning of the training program and at the end of the route.

**TABLE 1:** Descriptive analyses of psychological and motor tests of the entire sample (*n* = 12).

	Mean (T0 | T1 | T2)	*SD* (T0 | T1 | T2)	Min (T0 | T1 | T2)	Max (T0 | T1 | T2)
Bend and reach	−17.9 | 19.3 | 18.8	17.2 | 11.9 | 11.8	−41 | 4 | 3	12 | 34 | 35
Queens college step test rest	86.2 | 84.4 | 87.6	9.01 | 7.65 | 12.1	74 | 71 | 65	104 | 100 | 104
Queens college step test effort	116 | 115 | 119	13.7 | 13.4 | 16.2	103 | 90 | 98	137 | 139 | 145
Deep squat	1.5 | 1.75 | 1.83	0.67 | 0.62 | 0.71	1 | 1 | 1	3 | 3 | 3
In-line lunge test	2.08 | 2.50 | 2.58	0.79 | 0.52 | 0.51	1 | 2 | 2	3 | 3 | 3
Shoulder mobility (left)	−13.8 | −9.5 | −6.5	14.2 | 7.56 | 8.26	−40 | −19 | −19	3 | 4 | 6
Shoulder mobility (right)	−12.8 | −6.17 | −2.58	20.7 | 7.81 | 6.89	−67 | −16 | −14	11 | 10 | 10
Shoulder mobility	2.25 | 2.42 | 2.58	1.06 | 0.66 | 0.51	0 | 1 | 2	3 | 3 | 3
Active straight leg raise	1.83 | 1.83 | 1.92	0.71 | 0.71 | 0.79	1 | 1 | 1	3 | 3 | 3
Rotary stability	2.17 | 2.58 | 2.92	1.11 | 0.51 | 0.28	0 | 2 | 2	3 | 3 | 3
FMS total score	9.83 | 11.1 | 11.8	2.95 | 2.19 | 1.90	6 | 8 | 9	15 | 15 | 15
STAI-Y1	35.64 | 35.08	6.31 | 12.99	27 | 21	47 | 67
STAI-Y2	44.18 | 39.67	9.69 | 14.64	28 | 24	62 | 67
BDI-II	9.18 | 4.92	7.08 | 6.29	1 | 0	21 | 18
PedsQL	13.95 | 22.50	5.09 | 10.24	4 | 6	21 | 45

*STAI-Y1, state anxiety inventory–Y form; STAI-Y2, trait anxiety inventory–Y form; BDI-II, beck depression inventory II; PedsQL, pediatric quality of life inventory 4.0 young adult version.*

We performed four one-tailed paired *t*-test to analyze the mean differences between scores collected before and after the activities. For the analysis, we considered the STAI-Y1 and STAI-Y2 scores, BDI-2, and PedsQL scores. Results showed that BDI-II scores were significantly lower (*p* < 0.05) after the entire program. Meanwhile, scores concerning the quality-of-life (PedsQL) significantly improved after the activity (for the test of quality-of-life *p* < 0.05). No significant differences were detected when considering state anxiety during the assessment. Indeed, trait anxiety was not statistically different before and after the program.

For the motor tests, we ran a repeated measures ANOVA test that compares means across one or more variables that are based on repeated observations. In our study, we tested participants 1 week before the beginning of the training route, 1 week before the 9 days route, and 1 week after the 9 days route. Results shown in Table 2 highlight statistical differences across means in the following test: bend and reach (*p* < 0.001), deep squat (*p* = 0.031), in line lunge test (*p* = 0.014), left shoulder mobility (*p* = 0.045), rotary stability (*p* = 0.015), and FMS total (*p* = 0.003). No differences were detected for Queens College Step Test in both conditions, shoulder mobility test and active straight leg raise test.

**TABLE 2:** Paired samples *t*-test for psychological measures and repeated measures Anova for motor tests.

Paired samples *T*-Test		Statistic		Df	*p*
STAI-Y1 (T0)	STAI-Y1 (T1)	0.181		11.0	0.860
STAI-Y2 (T0)	STAI-Y2 (T1)	1.076		11.0	0.305
BDI-II (T0)	BDI-II (T1)	3.192		11	0.009*
PedsQL (T0)	PedsQL (T1)	−2.928		11	0.014*

**Repeated measure anova test**	**Sum of squares**	**df**	**Mean square**	**F**	***p***

Bend and reach	10954	2	5477	20.0	<0.001*
Queen college step test rest	60.4	2	30.2	0.493	0.617
Queen college step test effort	129	2	64.4	0.569	0.574
Deep squat	0.722	2	0.3611	4.09	0.031*
In line lunge test	1.72	2	0.861	5.25	0.014*
Shoulder mobility (left)	318	2	159.2	3.59	0.045*
Shoulder mobility (right)	649	2	325	3.18	0.061
Shoulder mobility	0.667	2	0.333	0.786	0.468
Active straight leg raise	0.0556	2	0.0278	1.000	0.384
Rotary stability	3.39	2	1.694	5.12	0.015*
FMS total	24.5	2	12.25	7.74	0.003*

STAI-Y1, state anxiety inventory–Y form; STAI-Y2, trait anxiety inventory–Y form; BDI-II, beck depression inventory II; PedsQL, pediatric quality of life inventory 4.0 young adult version. *p < 0.05.

## Discussion

The innovations of this project were to implement in a real-life group context a structured activity of social skills usually carried out only on an outpatient basis and to treat structured motor training in a natural environment for an extended period. Addressing social deficits and physical health problems, especially motor deficits, in a real-life project clinically improved people with ASD social, physical, and mood difficulties. These results represent an interesting starting point for similar projects aiming to consider the person with a comprehensive perspective. In fact, projects like this can parallel work in a habilitative viewpoint on autism core symptoms and on ASD side symptoms.

The strength of this study was to allow the participants to generalize during everyday life the social skills acquired during training sessions. Furthermore, they received constant verbal prompts from trainers and social reinforcement from peers and trainers when they exhibited the skills learned. Trainers observed a qualitative clinically relevant social behaviors improvement and less severe stereotypies. Another important result of the project was to have the possibility to obtain more clinical information on the participants’ use of social skills in real-life compared with the outpatient setting. Likewise, being welcomed by the local authorities of most of the municipalities along the way, by senators and parliamentarians, the Minister for Disabilities from the Italian Republic, and by Pope Francis in a private audience represented a significant social reinforcement for the participants.

Participants enjoyed the project especially for being able to overcome their difficulties and improve their relationship with peers.

Finally, the results highlight the statistically significant improvement in quality-of-life and mood. These results are in line with other studies demonstrating the beneficial effect of physical training in reducing anxiety and depression (28, 29). The statistically significant results of exercise as a treatment for mild-to-moderate depression provide support to the notion that a cognitive-behavioral intervention can have a substantial effect on mental wellbeing.

For what concerns state anxiety, this was not statistically different before and after the route, indicating that contingent anxiety effects did not affect the compilation of the questionnaire. However, in the case some uncontrolled anxiety effects had been present during the compilation, the intensity was presumably the same in both experimental conditions.

Aside from the promising results of this initiative, sharing images of the journey in real-time on Facebook and after with the film represented a possibility to spread a positive image of autism, of people who, regardless of their difficulties, walked, lived, and above all had fun together, to fight against the stigma of disability.

## Data Availability Statement

The original contributions presented in the study are included in the article/supplementary material, further inquiries can be directed to the corresponding author/s.

## Ethics Statement

The studies involving human participants were reviewed and approved by the Regione Piemonte, ASL Città di Torino. The patients/participants provided their written informed consent to participate in this study.

## Author Contributions

RK contributed to conception and design of the study and wrote the manuscript. FA, GC, and EN organized the database and performed the statistical analysis, and contributed to writing the manuscript. LS revised the manuscript. RK, FA, CF, RC, CG, and ML collected the data. All authors contributed to manuscript revision, read, and approved the submitted version.

## Conflict of Interest

The authors declare that the research was conducted in the absence of any commercial or financial relationships that could be construed as a potential conflict of interest.

## Publisher’s Note

All claims expressed in this article are solely those of the authors and do not necessarily represent those of their affiliated organizations, or those of the publisher, the editors and the reviewers. Any product that may be evaluated in this article, or claim that may be made by its manufacturer, is not guaranteed or endorsed by the publisher.
